# The Inherent Human Aging Process and the Facilitating Role of Exercise

**DOI:** 10.3389/fphys.2018.01135

**Published:** 2018-10-08

**Authors:** Norman R. Lazarus, Stephen D. R. Harridge

**Affiliations:** Centre for Human & Applied Physiological Sciences, School of Basic & Medical Biosciences, Faculty of Life Sciences & Medicine, King’s College London, London, United Kingdom

**Keywords:** aging, exercise, performance, healthspan, physiology

## Abstract

Arguably the best available depictions of the global physiological changes produced by age are the profiles of world record performance times in swimming, athletics, and cycling, depicting the trajectory of decline in maximal integrated physiological performance capability. The curves suggest that the aging process produces a synchronized, controlled decrease in physiological performance over the human lifespan. The shape of the performance profile by age is essentially independent of discipline, distance, or phenotype. Importantly, the specific times of performance are not the driving force in the production of the shape of the declining performance profile. We suggest that in these highly trained individuals the shape of the curve is generated by the aging process operating on a physiology optimized for any given age. We hypothesize that with adequate training this same profile and trajectory, but with lower performance times, would be generated by all individuals who engage in sufficient physical activity/exercise. Unlike performance, data obtained from examining individual physiological systems or tissues do not give information on the unceasing and changing global integrating functions of the aging process. However, these data do give valuable information about the integrity of physiological systems at a particular age and allow a direct comparison to be made between the effects of inactivity and physical activity/exercise. Being physically active has been shown to have global protective effects on physiological systems and thus facilitates the aging process by maintaining physiological integrity. There is emerging evidence which suggests that physiological regulation of aging may be multi-compartmentalized. We do not advocate exercise as a panacea, but all the evidence indicates that being physically active and exercising is far superior to any other alternative for achieving optimal aging.

## Introduction

The aging process is generally described as being closely associated with the onset of disease. Yet there is an extensive literature documenting that many of these diseases of aging are heavily influenced by lifestyle factors, namely physical inactivity, exercise and diet. Readers are referred elsewhere for more expansive definitions of physical activity and exercise (Centers for Disease Control and Prevention, 2014). It is also becoming clear that not only is the expending of energy (through physical activity or exercise) *per se* important, but that the time spent being sedentary, and especially time spent sitting, is also highly deleterious to physiological function and health across the lifespan (Bouchard et al., 2015). In this review the majority of the discussion and evidence accessed from the literature is based on the influence of exercise on aging, i.e., those that have undertaken planned, structured, repetitive physical activity that is purposeful in the sense that the objective is an improvement or maintenance of one or more components of physical fitness (Centers for Disease Control and Prevention, 2014). The diseases which relate to a lack of exercise, low levels of physical activity and sedentary behavior include; include sarcopenia, metabolic syndrome, obesity, insulin resistance, type 2 diabetes, non-alcoholic fatty liver disease, coronary heart disease, peripheral artery disease, hypertension, congestive heart failure, endothelial dysfunction, deep vein thrombosis, depression and anxiety, osteoporosis, osteoarthritis, rheumatoid arthritis, as well as a number of different types of cancer including colon, breast, and endometrial cancer (Booth et al., 2014; Pedersen and Saltin, 2015).

The descriptions of the interactions of exercise, diet and health have been common place in textbooks of exercise physiology for many years (e.g., Åstrand and Rodahl, 1986; Blair, 2001; McArdle et al., 2006). Yet whilst the responses and adaptations to exercise on physiological systems are well documented, the aging process itself remains an enigma. Healthy aging appears in the context of exercise physiology, but still is largely omitted as a core part of the medical curriculum, as is evidenced by a perusal of many medical textbooks. Surprisingly, there is also relatively little reference to the effects of exercise on the etiology of diseases of aging and more of a focus on the therapeutic effects of exercise on a wide range of aging related diseases (ACSM: Guidelines for Exercise testing et al., 2014; Pedersen and Saltin, 2015). In recent years there has been a movement to include exercise in therapeutic regimens (Hallal and Lee, 2013), even though the effectiveness of exercise in the treatment of heart diseases has essentially been implied since the seminal work of Morris et al. (1953). The current majority approach to the treatment of the preventable diseases associated with inactivity (e.g., cardiovascular disease, obesity, type 2 diabetes) is to rely on pharmaceutical therapy after the onset of the disease (Partridge, 2014). Exercise may only then be prescribed to correct the physiological deficit caused by the inactivity and energy imbalance. One of the unfortunate outcomes arising from ignoring prevention is demonstrated in a large cohort of individuals in the United Kingdom followed for 70 years, where 75% were on pharmaceutical therapy (Pierce et al., 2012). It has long been argued that this pharmaceutical approach to aging results in multi-pharmaceutical medication as one system failure follows another (Estes and Binney, 1989; Gems, 2011).

The aim of this short review and commentary is to try and present a different perspective of the aging process, emphasizing the global nature of its effects on physiological function and how these effects are modified over time by lifestyle factors and by exercise in particular.

## The Nature of the Inherent Aging Process

Because aging probably effects every system and cell in the body, the obvious way of measuring these effects would be by having an indicator that accurately reflects the relationship between aging and the changes in physiological function that are occurring over a period of at least eight decades. Indicators of all-cause mortality are generally associated with changes in physiological function that can be modified by exercise. The indicators are therefore malleable. The result of this malleability raises a number of important points. The first is that when, for example, the maximal rate of oxygen consumption (VO_2max_) of exercisers and non-exercisers of the same age are compared, the exercisers will have a higher VO_2max_ than non-exercisers (Wilson and Tanaka, 2000; Lazarus and Harridge, 2010; Harridge and Lazarus, 2017). The link between a specific age and a specific value for that age will have been broken in a widely accepted marker of all-cause mortality (Blair et al., 1989). Secondly, both in exercisers and non-exercisers it is found that the same VO_2max_ value occurs in subjects differing by two or three decades (Harridge and Lazarus, 2017). The exercisers will have higher values than their non – exercising counterparts. Again, the link between a specific age and a specific VO_2max_ value will have been broken. These facts demonstrate not only malleability, but that exercisers and non – exercisers have different phenotypes. The concept of a physiological marker that appears to be independent of the malleability of the function it is measuring is interesting. The recent paper by Levine et al. (2018) on epigenetic markers suggests such a marker. This needs to be rigorously and widely tested, particularly against defined populations of exercisers and non – exercisers.

**FIGURE 1:**
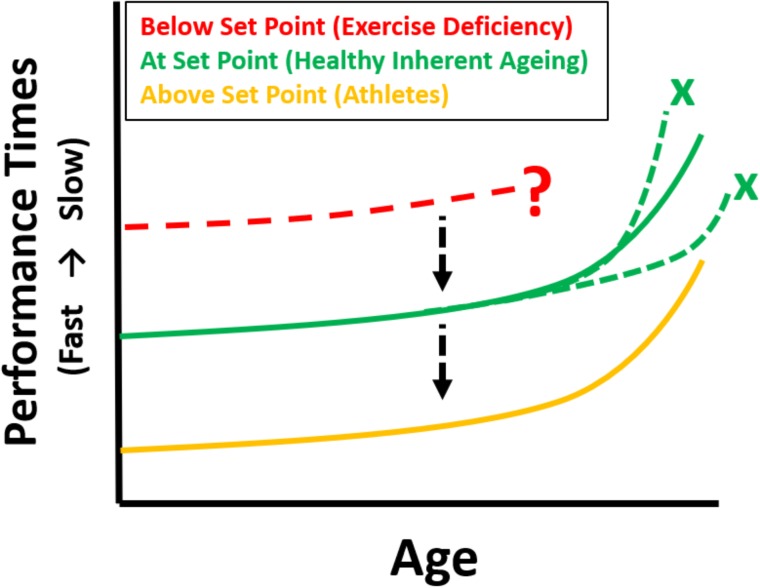
Theoretical longitudinal maximum performance curves (e.g., swimming, running, cycling) for athletes as they age [based on cross-sectional data from World Record performances, Baker and Tang (2010), gold]. Inherently aging individuals exercising at Set Point (green) are predicted to have the same trajectory of decline if they were to undertake a similar athletic event at regular intervals as they age (depicting the change in integrative whole-body physiology due to aging), but with inferior performances. Maintaining the same profile, but improving athletic performance is possible by increasing exercise towards the gold zone. Those below set point show an unpredictable trajectory (red). The black arrows indicate the change to different performance curves with increased levels of physical exercise, the green crosses indicate that if exercising at set point there can be no acceleration or deceleration (i.e., deviation from the predicted curve which represents inherent aging) – assuming no disease. The red dotted curve and question mark indicate both an uncertain trajectory, or indeed the very possibility, of being able to undertake a maximal performance.

It appears that aging affects nearly all physiological systems. In order to follow these changes a measure of global physiological function is necessary. Such a measure is maximal athletic performance where many systems must be integrated to be able to perform at their limits. Thus, a change in performance over time (years) reflects the change in global physiological status with aging. Such performances are depicted in the records of Master Athletes (Baker and Tang, 2010; Lazarus and Harridge, 2017) and in these highly trained individuals, the negative effects of inactivity are completely removed. We suggest that these performance profiles (Gold curves depicted in **Figure 1**) are generated by the effects of the aging process because the following important variables are controlled: (1) all competitors are at maximal physical effort in order to produce best times, (2) the intensity, type and frequency of training will be similar, (3) the type of exercise is the same for all competitors, (4) lifestyle and dietary habits are geared to maximum performance, (5) the ages of the entrants in any championship event cover the human lifespan, (6) performance times are objectively measured and (7) in any single discipline phenotype is probably going to be as homogeneous as can be expected in cross-sectional studies. Thus, confounding variables such as healthcare, nutrition, socio-economic conditions between generations, as well as genetic differences between individuals are ameliorated.

The profiles produced by aging runners, swimmers and cyclists all follow the same age- related decrease in performance (Donato et al., 2003; Baker and Tang, 2010). The decline in performance is curvilinear tending to accelerate after the seventh or eighth decade. Even more surprising is that the time course is similar in both sprint and endurance events, in other words independent of time of performance and phenotype (Lazarus and Harridge, 2017). The mechanisms whereby the aging process produces superimposable performance profiles from differing phenotypes are unknown. In addition, these data need to be interpreted in the context of factors which include: (i) there is a shrinking pool of competitors in the older age groups, (ii) the fact that older world record holders are unlikely to been the champion athletes in their youth and (iii) master sports is not well developed in all countries.

**FIGURE 2:**
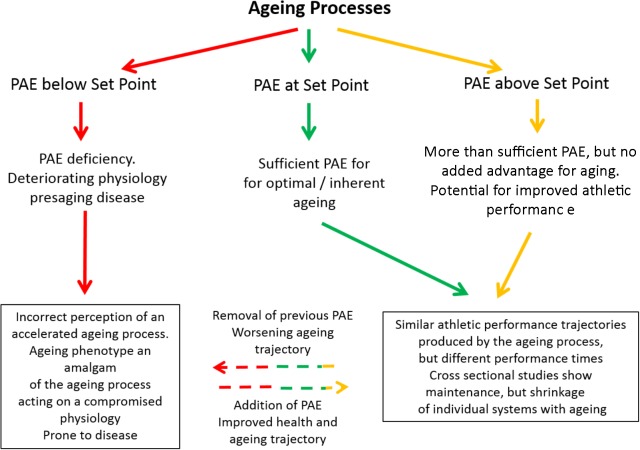
Schematic showing the effects of exercising below (red), at (green), or above (gold) the Set Point. Any protective effects of moving from gold/green to red are unclear (but overall effects are negative). The progression from red through to green will be an overall positive effect, whilst the progression from red through to gold and being able to fulfill athletic potential in later life is unclear. PAE refers to Physical Activity/Exercise.

It has been postulated that the exceptional performance times are partly due to the presence of “athletic genes” (Tucker and Collins, 2012) and therefore non-reflective of the ability of the general population who regularly engage in exercise. That may well be, but the main interest of this review is not in the performance times themselves. The more pertinent question is whether the shape of the performance profile generated by the aging process is a universal aging profile or is it only applicable to champion athletes? This question cannot be directly answered because, not surprisingly, there are no championships for average performers in any discipline. However, examination of swimming performance in Class six as defined by the Paralympic Committee (2015) may give some insight. This class shows that the competitors, although not conforming to the stereotypical phenotype of master swimmers, are capable of producing extraordinary performances. We hypothesize that with the same facilities, coaching and training advice, average swimmers, for example, would produce maximum times appropriate to their abilities and that the profile of decreasing performance as a result of age would be the same as that produced by better physically endowed athletes. This concept is demonstrated as the solid green curve in **Figure 2**.

Aside from hypothetical elite genes there are other related issues that need to be addressed. Firstly, do people engage in exercise because they have a genetic predisposition to respond to exercise, and secondly, do about 80% of people choose inactivity because they cannot respond to exercise? Unsurprisingly, in a cross-sectional study of non-competitive men and women cyclists (albeit again self-selected) it was found that all the physiological systems showed the expected effects of high levels of physical activity (Pollock et al., 2015). The Canadian Heritage studies demonstrate that there are about 20% of the population who will not respond appropriately to exercise (Bouchard, 2012). How the aging process operates in these individuals has yet to be investigated. Another population, the frail, represent a group whose symptomatology can be ascribed to an exercise deficiency (Lazarus et al., 2018). These patients, which represent the very antithesis of exercisers, are also able to exhibit a positive response to an exercise intervention program (Cadore et al., 2014; Maddocks et al., 2016). Sometimes personal choice is as important as genetics.

## The Set Point Hypothesis: Predictions and Conundrums

We (Lazarus and Harridge, 2017) have recently put forward the hypothesis that for optimal aging to occur, exercise must be at a level that equates to an individual’s “set point.” At set point physiological function is free of the effects of inactivity (**Figure 2**). The level of exercise necessary to reach set point will vary and is determined by individual differences in physiological ability. Operating on this platform of protected physiological function, the aging process is optimal. Thus, all subjects engaging in exercise at their set point will follow a pathway that results in optimal aging and decreased morbidity in later life. The hypothesis is now extended and suggests that if individuals, irrespective of whether they were at set point or above, could be tested for performance over a lifetime, then the effect of age would generate the same curvilinear-shaped decline in performance irrespective of times (**Figure 1**). Any increase in exercise above the built-in set point does not impinge on the aging process, but it does allow better performance times by expanding physiological capability (**Figure 2**). The inherent aging process generates the same age-related decline in performance for all who engage in exercise, provided other lifestyle factors such as smoking, alcohol intake and diet are controlled.

Some researchers use terms such as accelerated (Simon et al., 2006) or decelerated (Olshansky et al., 2007) aging. These terms can refer to changes in telomere length with shortened telomeres supposedly indicating accelerated aging. The terms are generally unfortunate because, whatever their basis, they give the impression that the inherent aging process can be manipulated. Instead, we suggest that aging be considered as having two modes of presentation. Either an optimal outcome that results from the inherent aging process operating on a diminishing, but fully primed physiology, or an uncertain outcome resulting from the aging process operating on a diminished and compromised physiology which is the result of inactivity and other lifestyle factors. The shape of each performance curve is independent of ability and is probably driven by the inherent ageing process. Thus, in theory no deviations in the shape of a curve are allowable (dotted green lines in **Figure 1**) for the disciplines mentioned (e.g., running, swimming, cycling). It remains to be seen if in future years and with more competitive athletes whether the trajectory of change predicted by world records will alter. If it is representative of the aging process then this shape will remain essentially the same.

If the age-dependent change in performance time follows the same curve for all exercisers, irrespective of ability, discipline, distance, or phenotype then do these curves all intersect baseline at the same age? In other words, would all exercisers die at round about the same age? There is some evidence that elite athletes have greater longevity than the general population (Teramoto and Bungum, 2010; Garatachea et al., 2014). This is unsurprising because most of the general population do not engage in sufficient physical activity or exercise and so are prone to all the diseases of inactivity and their consequences (Booth et al., 2014). We are unaware of any longevity data comparing the age of death of athletes from different disciplines. An initial study showed that male athletic champions had a significantly lower mortality than the general population under the age of 50 years; after 50 years of age the mortality was the same (Schnohr, 1971). A more recent systemic review of 54 studies reported superior lifespan longevity outcomes for elite athletes compared to age- and sex-matched controls from the general population and to other athletes (Lemez and Baker, 2015). In a study of 393 former male Finnish elite level athletes (∼72 years), a lower body fat percentage, lower risk for the metabolic syndrome and non-alcoholic fatty liver disease was reported (Laine et al., 2016). Having had a career as an elite-class athlete during young adulthood seemed to offer some protection, but current exercise levels and volume of leisure time physical activity were suggested to be equally important factors. This is predicted by the set point theory. For example, if exercise is markedly reduced after 50 years of age then the athletes would likely revert to the same inactivity-compromised physiology as the general population. Perhaps the most relevant question is whether elite athletes live longer than those subjects who engage in exercise at their set point? There is no information, but our hypothesis states that exercise above set point confers no additional health benefit; therefore, the longevity of these latter subjects should be equivalent to elite athletes. However, longevity is arguably not the most appropriate index to follow. For the elderly, being free from disease and being independent is probably more important than longevity. This independence is reflected by the “healthspan” (Fries, 1980; Seals et al., 2016). Healthspan is optimal at set point and above assuming no over-training pathologies.

Whilst conferring health benefits, exercise above the set point and engaging in very high levels of exercise can carry its own risks. These relate to musculoskeletal wear and injury (Kujala et al., 2005) as well as overtraining suppression of the immune system (Gleeson et al., 1995).

## Cross-Sectional Studies: Aging, Physiological Function, and Exercise

Cross-sectional studies, that do not measure performance, allow for a limited but important understanding of the effect of exercise on the aging process. These studies allow comparison of the effects of exercise versus inactivity on physiological systems across the human age spectrum but give no information about the integrating functions of the aging process. This rarely acknowledged deficiency in the interpretation of cross-sectional data has had a profound influence on the perception of aging especially if inactive subjects with compromised physiologies have been used as examples of healthy aging in aging research. For example, it has been reported that mitochondrial function declines with age (Gonzalez-Freire et al., 2018), yet there was no objective assessment of the individuals exercise behavior. The heterogeneity in the results also makes it impossible to ascribe any one level of mitochondrial function to a specific age. Will the same physiological index value suffice for a 70-year-old exerciser and a 70-year-old inactive person? The available data indicate probably not (Wilson and Tanaka, 2000; Pollock et al., 2018). We have previously discussed the issue regarding the relationship (or lack of) between age and function across multiple physiological indices (Lazarus and Harridge, 2010, 2011; Harridge and Lazarus, 2017). Researchers, whatever their field, can thus be prone to incorrectly ascribing changes in physiological indices as being due to age instead of the result of the aging process operating on an inactivity compromised physiology. This lack of appreciation of the interactions between aging, disease and exercise medicalises aging and often wrongly favors pharmaceutical and other treatments over preventive measures (Lazarus et al., 2018).

## Effects of Exercise on Physiological Systems and Layers of Regulation During Aging

**FIGURE 3:**
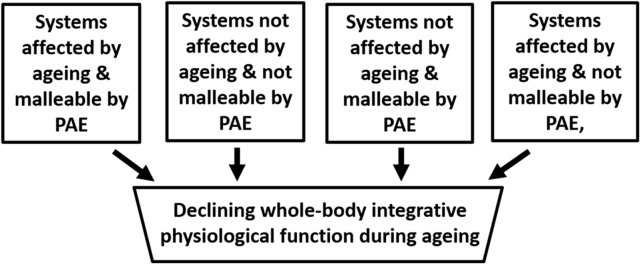
Schematic depicting the compartmentalisation of factors that impact on aging. The assumption here is that no pathologies are present. PAE refers to Physical Activity/Exercise.

Cross-sectional studies have shown exercise to have a positive effect on many physiological functions and there is an extensive literature on the subject that need not be re-iterated here (Booth et al., 2014). However, as is depicted **Figure 3** physiological systems can be divided into four categories, which in the absence of pathology could be described as those that are highly sensitive to and interact with exercise (e.g., skeletal muscle), those that are independent of both exercise and aging (e.g., absorptive function of the small bowel), those that are not malleable by exercise are age dependent (e.g., maximum heart rate) and those that are not affected by age, but are malleable by exercise and physical activity (e.g., resting heart rate).

In an extensive study, the immune cellular profile and affiliated hormones of a cohort of cyclists aged 55–79 years were compared with both a healthy young group and a group of clinically heathy older people who did not undertake regular physical exercise (Duggal et al., 2018). The prevailing view is that the thymic microenvironment undergoes architectural and phenotypic changes with age including: loss of stromal epithelial niches, reduced numbers of thymic epithelial cells, replacement of lymphoid tissue with adipose tissue reducing active areas of thymopoiesis (Dixit, 2010; Chinn et al., 2012). However, in this study thymic function and output were maintained in the cyclists with high levels of exercise. The frequency of naïve T cells and recent thymic emigrants were both higher in cyclists compared with inactive elders. The cyclists also had significantly higher serum levels of the thymoprotective cytokine IL7 and lower IL6, which promotes thymic atrophy. Interestingly, the cyclists also showed evidence of reduced immune senescence, namely lower Th17 polarization and higher B regulatory cell. Markers of chronic low-grade inflammation (“inflamaging”), IL-6 and TNFα, were also markedly lower in the cyclists compared to the older non-exercisers and only slight higher than the young. However, the study also showed that CD28^-ve^ CD57^+ve^ senescent CD8 T cells did not differ between cyclists and the older non-exercisers (Duggal et al., 2018). Thus, these cell types may be added to the exercise-independent, but age-dependent category in **Figure 3**.

This study demonstrates that not only between different systems, but within a single system, the regulation of physiological function during aging may be compartmentalized and multi-layered (Harridge and Lazarus, 2017). Furthermore, there is an intriguing relationship between skeletal muscle, the most metabolically sensitive tissue to exercise and the thymus. In culture thymic cells express contractile proteins and can form myofibers and animal data suggest that changes in the density of thymic myoid cells may accompany acute and chronic demands for muscle precursors (Wong et al., 1999). Thymic cells have also been suggested for myoblast transfer (Pagel et al., 2000). Could there be other links in active humans? Certainly, the maintenance of thymus function in the presence of continued skeletal muscle activity suggests molecular or hormonal links that need to be explored.

Undoubtedly, a global function like maximum athletic performance requires an intact immune system (Fitzgerald, 1998). However, performance unfortunately does not inform on the whole gamut of functions of the immune system. It may be that an intervention directed specifically at testing the immune system may be more appropriate. For example, a challenge by a specific antigen such as a flu antigen may be a better option. In this regard an exercise intervention in which participants were examined for improvements of the immune response to influenza vaccination showed that exercising participants had a greater increase in antibody and IFNγ production compared to less active controls (Kohut et al., 2005). However, unless the physical activity and exercise levels of the participants are reported, the results will provide little useful information for determining the global differences between the immune systems of those who are physically active and those that are not (Prezemska-Kosicka et al., 2018). We caution against the use of inactive subjects to confirm some of these associations.

## Some Final Thoughts on Regulation in Complex Biological Systems

The mutual interaction of the aging process and exercise ensures that the functional ability of the diminishing physiological base is kept as efficient as possible. The aging process accomplishes this effect by, as yet, unknown integrating and synchronizing mechanisms. We hypothesize a central location/s for the control of these integrating functions, but more data is necessary and at present our hypothesis is heuristic. There are other hypotheses concerning the control of complex systems. Many of these concern non-biological systems and will not be discussed. Kitano (2002) in his review of systems biology makes the point that “many breakthroughs in experimental devices, advanced software, and analytical methods are required before the achievements of systems biology can live up to their much-touted potential.” In the 16 years since this statement, there has been an exponential rise in the tools available in the field of systems biology, making advances in this field all the more possible. We would add other caveats; an understanding of the mechanisms whereby the aging process integrates and controls the declining *milieu interieur* over many decades is in its infancy. We hypothesize that as people, engaging in exercise, age-alternate pathways of regulation may be brought into play in order to keep shrinking physiological functions as optimal as possible. There is some evidence that in inactive, aging individuals there are shifts in control mechanisms; however, these shifts are related to failing systems rather than toward the maintenance of physiological integrity (Seidler et al., 2010). These crucial differences in regulation between failing systems and co-ordinate healthy aging should in no way be equated. As this brief review strongly suggests the physiology of aging is unfinished business.

## Author Contributions

Both authors contributed equally to the work and approved it for publication.

## Conflict of Interest Statement

The authors declare that the research was conducted in the absence of any commercial or financial relationships that could be construed as a potential conflict of interest.
